# Pulse wave analysis measurements: important, underestimated and undervalued parameters in cardiovascular health problems

**DOI:** 10.3389/fcvm.2023.1266258

**Published:** 2023-11-02

**Authors:** Philip Jan Claessens, Ruth Peeters, Louis Claessens, Christophe Claessens, Jan Claessens, Philip Maria Claessens

**Affiliations:** ^1^ UAntwerp, University Antwerp, Antwerp, Belgium; ^2^ UHasselt, University Hasselt, Hasselt, Belgium; ^3^ KU Leuven, Catholic University of Leuven, Louvain, Belgium; ^4^ UGhent, University Ghent, Ghent, Belgium; ^5^ Medical Center “Kloppend Hart Essen-Antwerpen- Schilde”, Antwerp, Belgium; ^6^ Department of Cardiology, AZ Monica Deurne, Antwerp, Belgium; ^7^ Department of Cardiology, AZ Voorkempen Malle, Antwerp, Belgium

**Keywords:** pulse wave analysis, arterial stiffness, hypertension, coronary atherosclerosis, valvular heart disease, ADHD

## Abstract

**Background:**

Central aortic stiffness is established as a reliable measure of cardiovascular disease. While pulse wave velocity (PWV) analysis measures arterial distensibility, risk profile of cardiovascular diseases can be expanded with following pulse wave analysis measurements: central aortic systolic blood pressure (CABPS), central aortic pulse pressure (CAPP), central aortic reflection magnitude (CARM), central aortic augmented pressure (CAAP) and central aortic augmentation index (CAAIx). The aim of this study is to evaluate the clinical usefulness and importance of pulse wave analysis measurements in specific cardiovascular conditions and diseases, both in term of diagnosis and therapeutic monitoring.

**Methods:**

One thousand sixty-six subjects were included. According to age bracket, four cohorts were investigated—healthy subjects (NL), hypertensive patients (HP), ischemic heart disease (IHD) and valvular heart disease (VHD) patients. Arterial stiffness was analyzed through Sphygmocor XCEL Central Blood Pressure Measurement System and Sphygmocor XCEL PWV Measurement System. Furthermore we observed the pulse wave analysis measurements of 14 patients with diagnose of ADHD who were referred by a child psychiatrist, in order to investigate the initiation of methylphenidate treatment.

**Results:**

Statistically significant differences were found between NL and HP cohorts, across almost all age brackets, regarding pulse wave analysis measurements. In the risk stratification of arterial stiffness hypertension and especially central aortic blood pressure systolic (CABPS) seems a determining factor. Pulse wave analysis measurements for IHD and VHD cohort comparisons with NL counterparts, revealed non- statistically significant variations. Elevated CAAP, CAAIx and CARM within the youngest age group (0–10 years) in attention-deficit-hyperactivity-disorder (ADHD) patients warrant attention.

**Conclusions:**

Following such investigations, CABPS appears as a robust predominant factor in problems of arterial stiffness. Pulse wave analysis and PWV are important parameters in the evaluation and monitoring of arterial hypertension and cardiovascular diseases. There is a hypothesis that CAAP could be an important and even decisive parameter in the diagnosis of ADHD. Further investigation needed.

## Highlights

- Across all cardiovascular conditions, there is a progressive increase in arterial stiffness with age.
- Pulse wave analysis and carotid femoral pulse wave velocity are accurate and non-invasive methods for assessing arterial stiffness.
- Central aortic systolic blood pressure (CABPS) measurement is a highly robust determining factor in the occurrence of arterial stiffness.
- Abnormal pulse wave analysis measurements in ischemic and valvular heart disease can be related to higher central aortic blood pressure.
- Strikingly high central aortic augmented pressure (CAAP) and central aortic reflection magnitude measurements (CARM) within the 0–10 year age bracket warrant attention and further investigation. Central aortic augmented pressure (CAAP) could represent an important and even decisive parameter in the diagnosis of ADHD.

## Introduction

The quality of the arterial vascular wall system is of paramount importance for the health and well-being of cardiovascular patients and—by extension—for global human health. Central aortic stiffness is established as a reliable measure of cardiovascular disease.

Increased arterial stiffness is considered as one of the earliest stages of the atherosclerotic process. Carotid-femoral pulse wave velocity (PWV) is commonly used and widely accepted as an accurate and non-invasive method for assessing arterial stiffness (1).

While PWV is a direct measure of arterial distensibility, the risk profile of cardiovascular and cerebrovascular diseases can be expanded and supplemented with other pulse wave analytical measurements, such as central aortic systolic blood pressure (CABPS), central aortic pulse pressure (CAPP), central aortic reflection magnitude (CARM), central aortic augmented pressure (CAAP) and central aortic augmentation index (CAAIx), which represent more complex parameters—depending upon vascular elasticity, compliance and peripheral resistance (2).

Arterial wall rigidity is considered to be an independent predictor of all-cause and cardiovascular morbidity/mortality within several clinical settings. These include hypertension, overweight status/morbid obesity (3), diabetes, end-stage renal-disease, lipid disorders (4), elderly age, cerebrovascular events and cognitive decline (5), inflammatory rheumatic/autoimmune diseases, vasculitis (6) and viral infections such as COVID-19 (7).

The aim of this study was to determine and evaluate arterial wall stiffness through pulse wave analysis measurements, according to age brackets, and findings were compared across cardiovascular conditions.

## Methods

### Experimental design and patient profiling

Between September 2021 and November 2022, a total of 1,066 subjects/patients were included in this study, divided into 10 age brackets. Each age bracket was further subdivided into 4 groups, as follows:

- Group I: Normal healthy subjects (NL)
- Group II: Hypertensive patients without other underlying medical pathologies (HP).
- Group III: Patients with ischemic heart disease, consisting of severe 2-/3-coronary artery disease, treated with either stent implantation or surgical myocardial revascularization and without major valvular heart disease or specific antihypertensive medication (IHD).
- Group IV: Patients with hemodynamically important poly-valvular heart disease, without significant coronary atherosclerotic lesions and without specific antihypertensive medication (VHD). The demographic values per age bracket and diagnosis are presented in Supplementary Table 24.

All patients were ambulatory/self-supporting, and consulted for a cardiac investigation, consisting of a clinical cardiac examination, electrocardiography with stress-test, together with three dimensional (3D) echocardiography with speckle-tracking echocardiography and advanced cardiac 3D quantification. These diagnostic procedures were employed to definitively exclude the presence of arrhythmias and heart failure.

In order to evaluate arterial wall elasticity/stiffness, the Sphygmocor™ XCEL Central Blood Pressure Measurement System and Sphygmocor™ XCEL Pulse Wave Velocity Measurement System were employed.

Pulse wave analysis was performed identically for all study participants, placed in a seated position for a minimum of 10 min of absolute rest with the back and right arm supported throughout all measurements. All patients were examined at the same time and environmental conditions. The measurements were always taken at the end of expiration.

Regarding parameters for assessing arterial stiffness, this investigation relied on the measurements of cuff brachial blood pressure systolic (CBBPS), central aortic blood pressure systolic (CABPS), central aortic pulse pressure (CAPP), central aortic augmented pressure (CAAP), central aortic augmentation index (CAAIx), central aortic reflection magnitude (CARM) and pulse wave velocity (PWV). All such measurements are defined as follows:

- CABPS is the pressure that target organs such as the heart kidneys and the brains experience and with which they are confronted in their function. Central aortic blood pressure systolic (CABPS) is the maximum pressure during ejection.
- CAPP is the central aortic systolic pressure minus the central aortic diastolic pressure. CAPP readings >50 mmHg were found to predict cardiovascular disease (8).
- CAAP is the difference between the two pressure peaks during ventricular systole. In case of increased arterial stiffness, superposition of forward and reflected pressure waves cause an increase in CAAP (9).
- CAAIx is the ratio of central aortic augmented pressure to central aortic pulse pressure, expressed as a percentage. A high CAAIx indicates increased arterial stiffness and exacerbated risk of organ damage.
- CARM is the ratio of reflected pulse height (backward wave) to forward pulse height (forward wave), expressed as a percentage.
- PWV measurement was performed through a femoral pressure cuff placed on the right upper thigh and a tonometer placed on the right carotid pulse in order to obtain a pulse wave reading. This study employed the subtraction method, namely, measurement of the distance from sternal notch to cuff, together with the distance from carotid to sternal notch and femoral-to-cuff distance (10, 11).

All pulse wave analytical measurements were all based upon central aortic blood pressure readings.

Stress and anxiety appear to have an impact on pulse wave analysis measurements. The finding that stress plays an important role in pulse wave analysis measurements is supported by the consistent observation of heart rates above 100 beats per minute, even following a sufficient rest and acclimatization period. The LF/HF ratio was assessed in these patients, and it was consistently observed that stress patients exhibited a value exceeding 3, indicating a dominance of the orthosympatical nerve system over the parasympatical nerve sytstem. This can be seen as a sign of supplementary stress disorders. Consequently, additional 2–3 control examinations were warranted within these patients during this study. In these circumstances, only measurements with lowest heart rates were included in the study.

### Statistical analyses

A series of multivariate analysis of variance (MANOVAs) were conducted to compare the means of group I against all other groups (II, III and IV) across all differing age brackets for CBBPS, CBBPD, CABPS, CABPD, CAPP, CAAP, CAAIx, CARM and PWV readings.

The interaction effects were examined further by splitting the dataset by age bracket and through performing MANOVAs for the differing age brackets separately.

Post-hoc pairwise comparisons (with Bonferroni correction) on the estimated marginal means of both groups in each age bracket determined the nature of this interaction.

The tables and interaction graphs highlight the comparison between the means (per parameter) between NL and HP, NL and IHD together with NL and VHD groups across the differing age brackets.

## Results

The multivariate outcomes demonstrated significant major effects for diagnosis (Pillai's Trace = 0.26, *F* = 10.79, *df* = 27, *p* < .001) and for age bracket (Pillai's Trace = 0.455, *F* = 6.11, *df* = 81, *p* < .001). The main effect of diagnosis indicated a difference in the overall means of the differing parameters between group I and group II, and the main effect of age bracket indicated a difference within overall means across the differing age brackets.

Consequently, the interaction between diagnosis and age bracket was significant; Pillai's Trace = 0.076, *F* (153) = 4.636, *p* < 0.001.

In the following sections we will discuss the post-hoc comparisons of next measurements: cuff brachial blood pressure and central aortic blood pressure, pulse wave velocity, central aortic pulse pressure, central aortic augmented pressure, central aortic augmentation index and finally central aortic reflection magnitude.

### Cuff brachial blood pressure (CBBPS and CBBPD)/central aortic blood pressure (CABPS and CABPD) readings

Upon probing arterial wall stiffness and cardiovascular diseases, this study analyzed four patient groups, each subdivided into 10 age brackets. These consisted of: 513 NL subjects; 322 HP patients; 136 IHD patients; 95 VHD patients.

**Figure 1:**
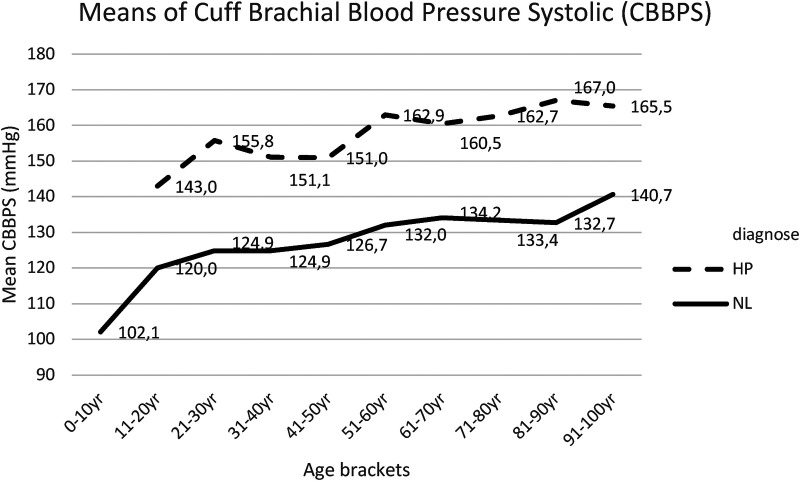
Mean CBBPS for each age bracket within NL and HP groups; NL, normal healthy subjects; HP, hypertensive patients.

**Figure 2:**
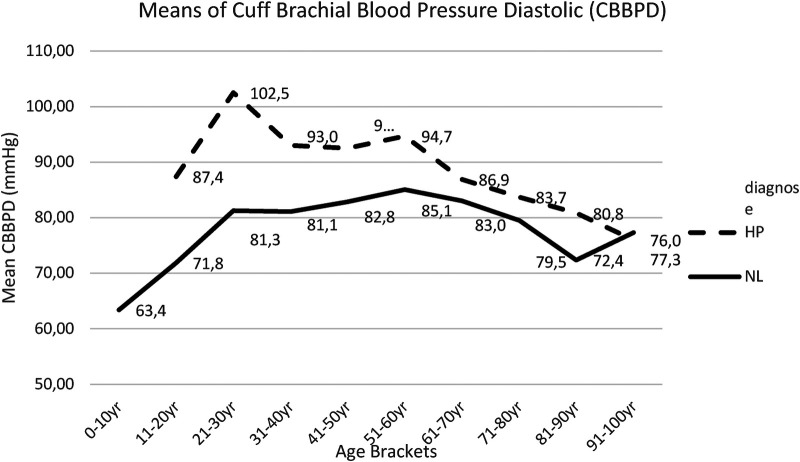
Mean CBBPD for each age bracket within NL and HP groups; NL, normal healthy subjects, HP, hypertensive patients; CBBPD, cuff brachial blood pressure diastolic.

Post hoc comparisons employing *t*-test with Bonferroni correction indicated that the mean score for CBBPS and the mean score CBBPD were significantly lower in NL compared with HP cohort over all age brackets, except for the 91–100 year age bracket. The results for CBBPS are presented in Supplementary Table 1 and in Figure 1, while results for CBBPD are presented in Supplementary Table 2 and Figure 2.

Concerning the cuff brachial diastolic blood pressure, this study reported a slight increase in both group I and group II, up to the age of 50 years, and a progressive decrease thereafter. Here too, this study maintained statistically significant differences between both groups, though less pronounced (Figure 2, Supplementary Table 2).

**Figure 3:**
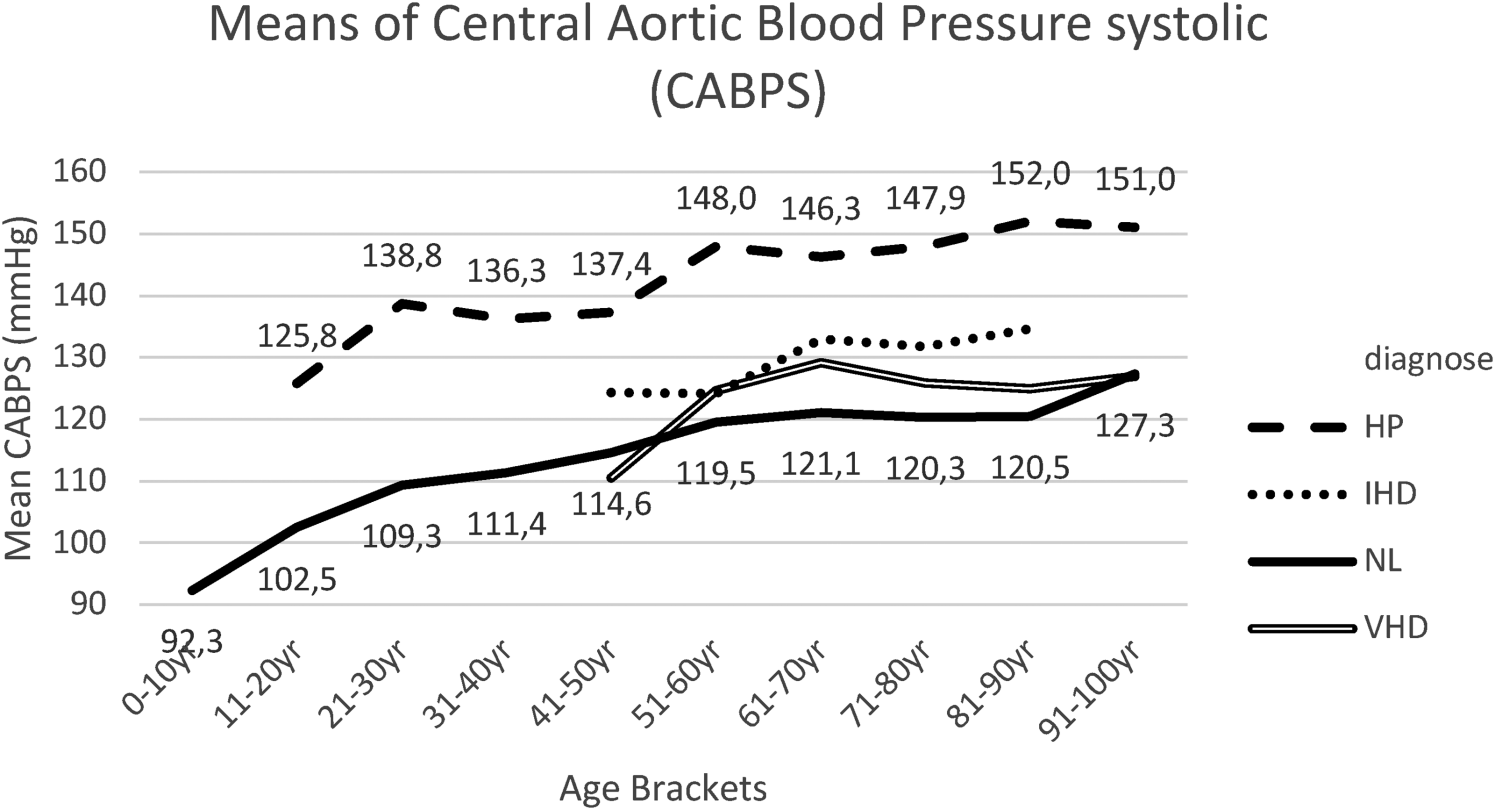
Mean CABPS for each age bracket within NL and HP groups. NL, normal healthy subjects, HP, hypertensive patients; IHD, ischemic heart disease, VHD, valvular heart disease; CABPS, central aortic blood pressure systolic.

**Figure 4:**
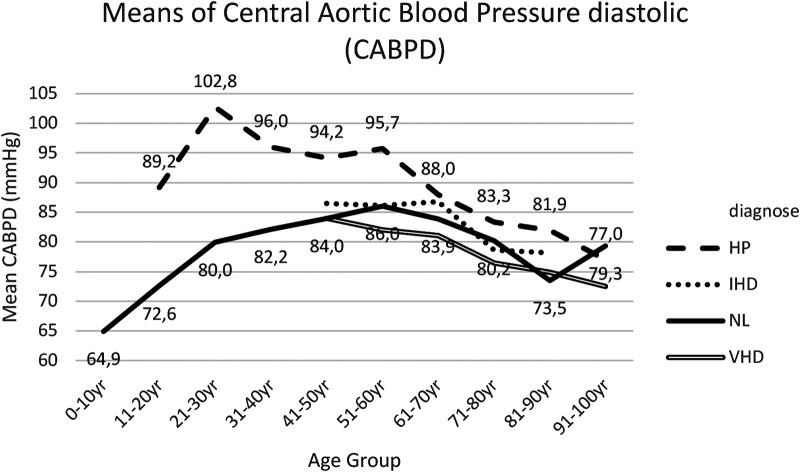
Mean CABPD for each age bracket within NL and HP groups. NL, normal healthy subjects, HP, hypertensive patients; IHD, ischemic heart disease, VHD, valvular heart disease; CABPS, central aortic blood pressure diastolic.

Similar findings were recorded for central aortic blood pressure measurements, both systolic (CABPS) (Figure 3, Supplementary Table 3), and diastolic (CABPD) (Figure 4, Supplementary Table 4).

The pairwise comparisons (with Bonferroni correction) for CABPS between group I and group II were significantly different for the age bracket 51–60 years till the age bracket 81–90 years, in which the means of group I were lower than the means of group II (Figure 3, Supplementary Table 3).

When comparing the means for CABPD between group I and group II, significantly different results were obtained in all age brackets, except in age bracket 71–80 years and age bracket 91–100 years (Figure 4, Supplementary Table 4).

Within group III, IHD patient cohort, CABPS and CABPD readings appeared slightly elevated compared to group I (NL), both without statistically significant differences (Figures 3, 4, Supplementary Tables 5, 6).

Within group IV, VHD patient cohort, this study noted slightly increased CABPS readings compared to group I (NL), though without statistically significant differences (Figure 3, Supplementary Table 7).

Slightly reduced—though statistically not significant—differences between CABPD readings were observed for group IV (VHD) compared to group I (NL) (Figure 4, Supplementary Table 8).

Main clinical signs of systemic hypertension in elderly patients are the divergent changes in central aortic systolic blood pressure and central aortic diastolic pressure, resulting in an increased CAPP, termed as isolated systolic hypertension, which is inherently associated with increased aortic stiffness (12).

### Pulse wave velocity (PWV)

**Figure 5:**
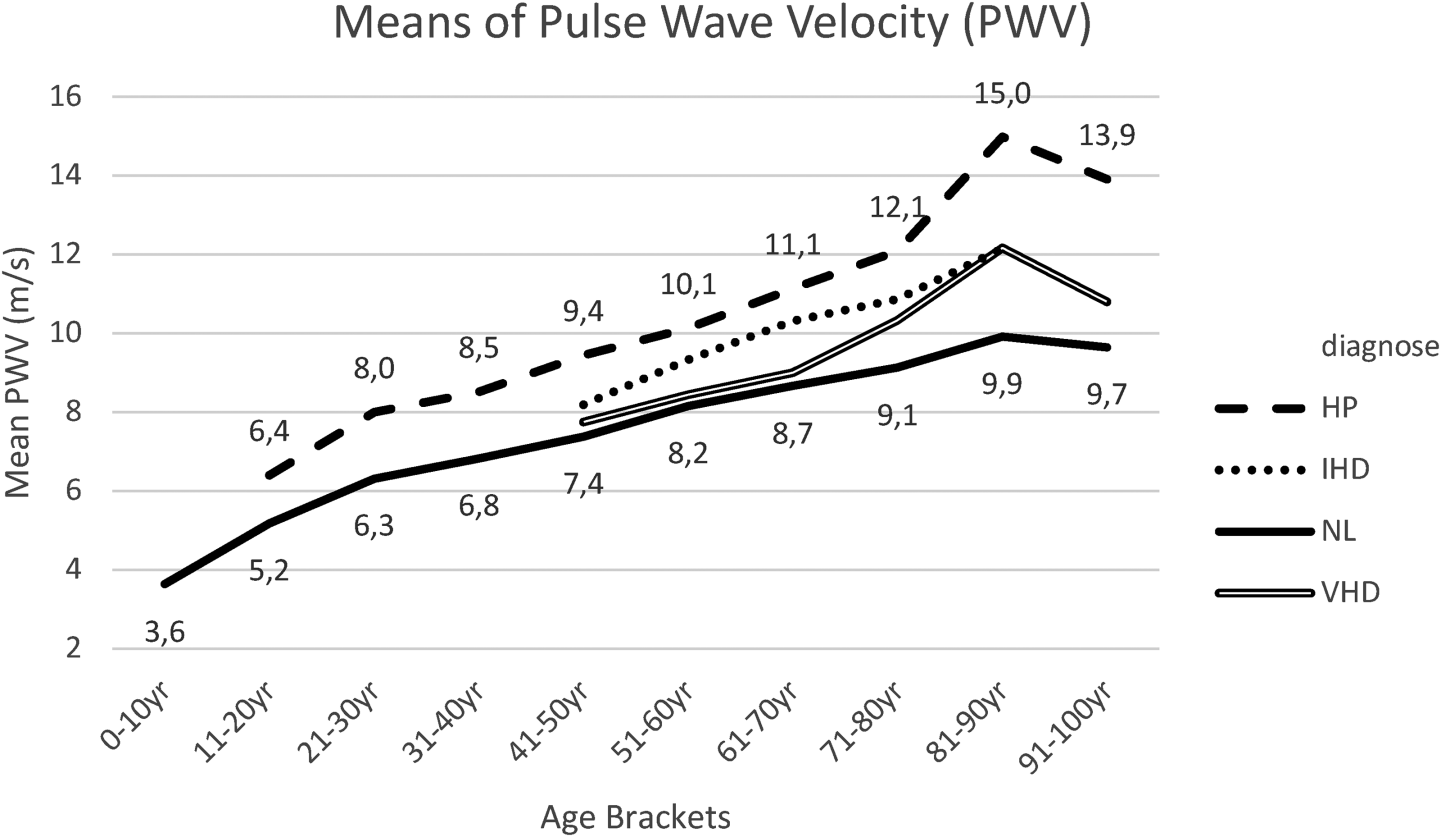
Mean PWV for each age bracket within NL and HP groups. NL, normal healthy subjects, HP, hypertensive patients; IHD, ischemic heart disease, VHD, valvular heart disease; PWV, pulse wave velocity.

Within Supplementary Table 9, the *post hoc* tests (*t*-tests with Bonferroni correction) for PWV means between group I (NL) and group II (HP) are presented. Significant results were obtained for the 51–60 years, 61–70 years, 71–80 years and 81–90 years age brackets, in which the mean PWV was lower for group I in comparison with group II (Figure 5).

Within IHD patients, this study recorded aortic PWV measurements, similar to—and in parallel with—NL and HP groups, although not revealing statistically significant variations (Figure 5, Supplementary Table 10).

Within VHD patients, this study noted a slight non-significant increase in PWV, compared to the NL- group, mainly occurring from 60 years and further across elder age brackets (16, 17) (Figure 5, Supplementary Table 11).

### Central aortic pulse pressure (CAPP)

**Figure 6:**
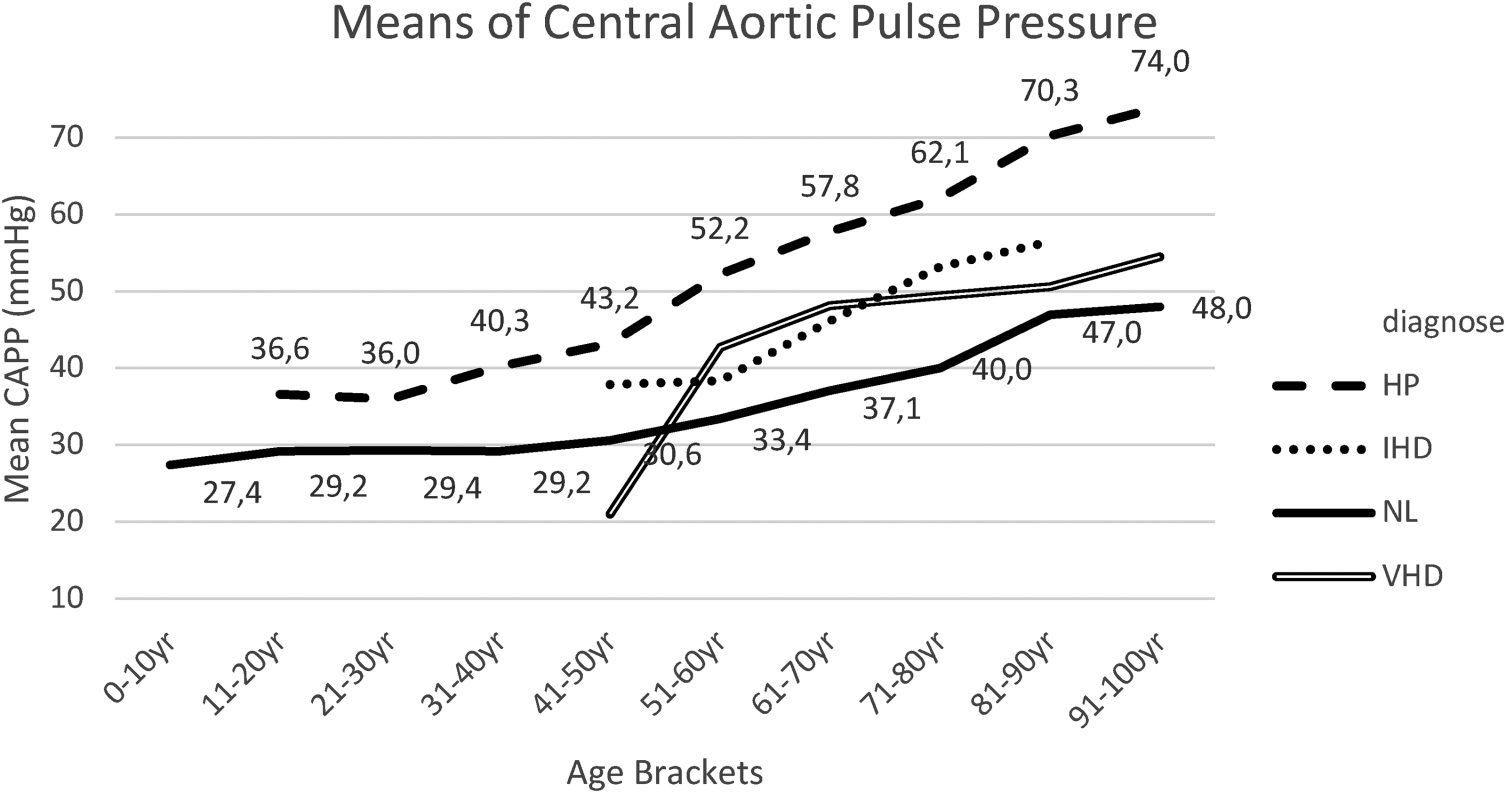
Mean CAPP for each age bracket within NL and HP groups. NL, normal healthy subjects, HP, hypertensive patients; IHD, ischemic heart disease, VHD, valvular heart disease; CAPP, central aortic pulse pressure.

Within Supplementary Table 12, the *post hoc* tests (*t*-tests with Bonferroni correction) for CAPP means between group I (NL) and group II (Hp) are presented. Results indicated that CAPP was lower in group I than in group II for the age bracket 31–40 years up until the age bracket of 91–100 years (Figure 6).

Within all groups, this study noted a progressive increase in CAPP according to age, with statistically significant differences between groups I (NL) and II (HP) from the age of 40 years, caused by divergent changes in central aortic systolic pressure and central aortic diastolic blood pressure, resulting in an increased central aortic pulse pressure (Figure 6, Supplementary Table 12).

When comparing patients of group III (IHD) and of group IV (VHD) with subjects from group I (NL), a slight increase in CAPP was noted with similar progressive course, according to age brackets, however without statistically significant differences (Figure 6, Supplementary Tables 13, 14).

### Central aortic augmented pressure (CAAP)

Within Supplementary Table 15, the *post hoc* tests for CAAP means between group I and group II are presented, whereby such results were significantly different for the age brackets of 31–40 years up to 81–90 years.

**Figure 7:**
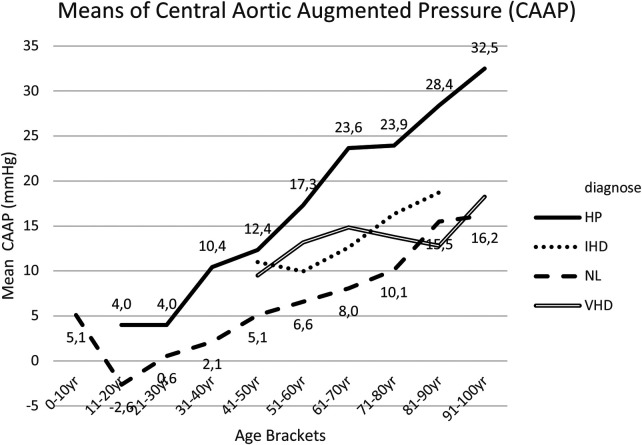
Mean CAAP for each age bracket within NL and HP groups. NL, normal healthy subjects, HP, hypertensive patients; IHD, ischemic heart disease, VHD, valvular heart disease; CAAP, central aortic augmented pressure.

CAAP increased linearly with age. Statistically significant differences between group I healthy subjects (NL) and group II hypertensive patients (HP), were progressively more pronounced within the older age bracket (Figure 7).

Compared to normal healthy subjects (NL), this study noted—in IHD and VHD cohorts—a slight increase in CAAP within the age brackets between 40 and 70 years, though not with statistically significant variations (Figure 7, Supplementary Tables 16, 17).

Furthermore, elevated CAAP readings within the age bracket 1–10 years were highly striking, which require our full attention (Figure 7).

The 14 patients in the age bracket 1–10 years, all demonstrated a normal cardiovascular examination and exercise capacity. However 10 patients were referred for cardiac examination with the diagnosis of ADHD syndrome, before starting a methylphenidate medication. The mean CAAP of the entire group was +4.71 mmHg; the mean for such ten ADHD patients was +6.3 mmHg, while in four patients without ADHD this was 0.5 mmHg. It seems that CAAP is a very decisive measurement in the diagnosis of ADHD.

The striking increased values of “central aortic augmented pressure” in otherwise apparently healthy children and in the young adults, in the absence of any underlying somatic pathology, require our further attention. This subject requires an attempt at interpretation and explanation, and potentially a more comprehensive control research, given the relatively small sample size of patients analyzed.

**Figure 8:**
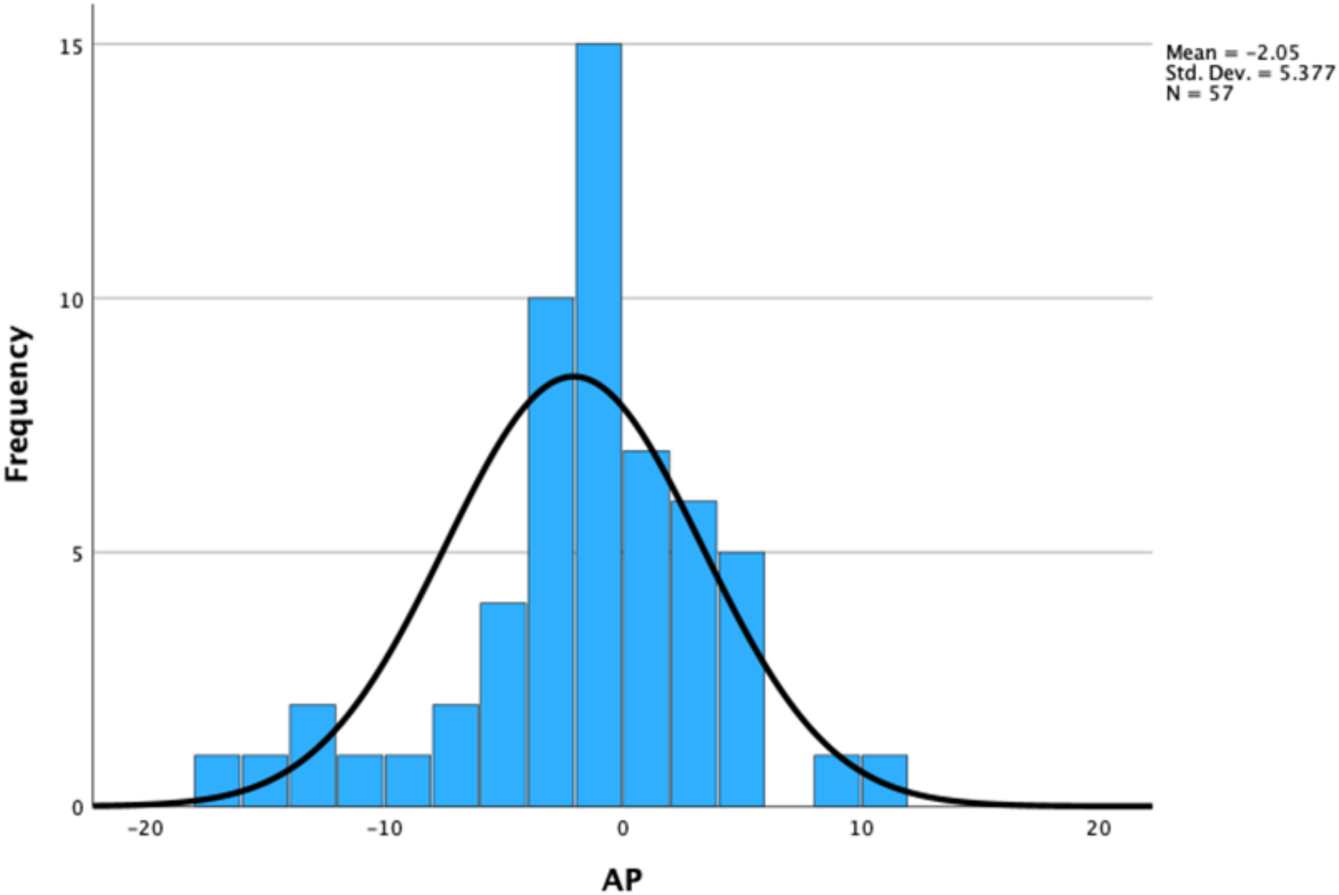
Confidence interval of CAAP within age bracket of 11-20 years.

Within the age bracket of 11–20 years, this study investigated CAAP in 69 normal healthy subjects (NL). Following 99% confidence interval testing, this study found an outlier with a CAAP value of +12 mmHg and a second outlier with a CAAP value of −19 mmHg (Figure 8).

**Figure 9:**
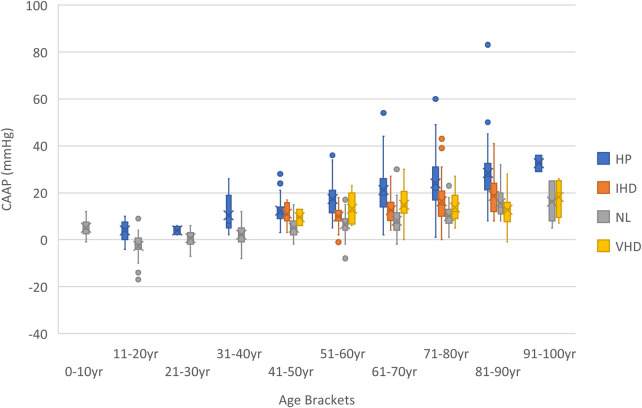
Boxplots of CAAP across all age brackets.

Boxplots of CAAP across all age brackets seem to indicate that cases of “possible supernormal vascular aging” appears to be limited to very young age brackets, while “early vascular aging” or “exaggerated vascular aging” was essentially only noticed within older age brackets (Figure 9).

### Central aortic augmentation index

**Figure 10:**
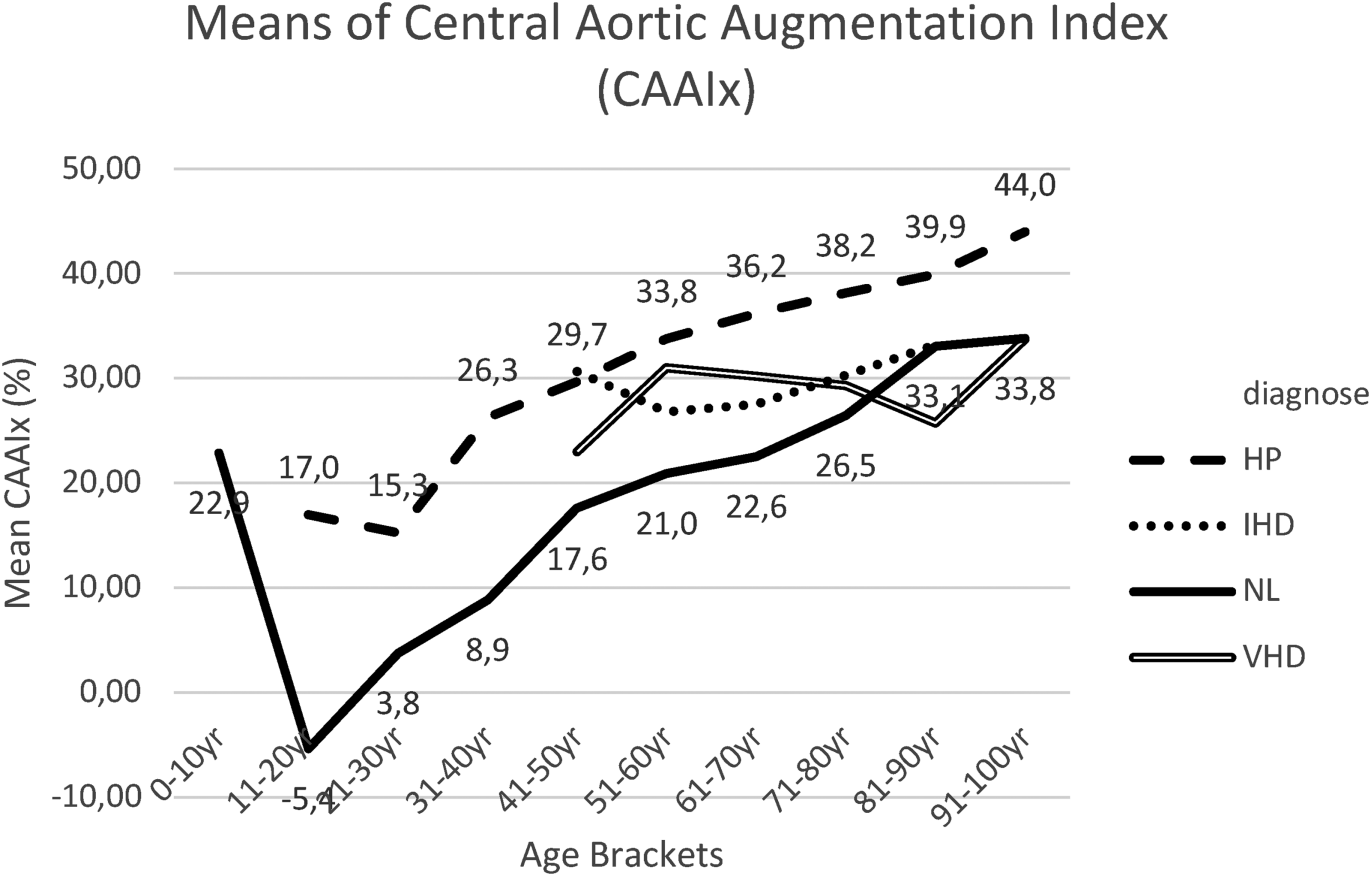
Mean CAAIx for each age bracket within NL and HP groups. NL, normal healthy subjects, HP, hypertensive patients; IHD, ischemic heart disease, VHD, valvular heart disease; CAAIx, central aortic augmentation index.

Figure 10 and Supplementary Table 18 present the results for *post hoc* tests for CAAIx means between group I and group II. These results indicated that the mean CAAIx was significantly lower within group I (NL) in comparison to group II (HP) across all age brackets, except for the last two age brackets (81–90 years and 91–100 years).

CAAIx progressively increased with age in both group I (NL) and group II (HP). Particularly within Group I, this study noted a steep rise of the curve up to the age of 50 years. Within younger individuals, CAAIx rose steeply with age up to 50 years, with further progressive—though more smooth and slower rise—up to 100 years of age.

In group III IHD patients (23) and in group IV VHD patients, CAAIx values appeared slightly increased compared to group I (NL), though without statistically significant variations (Figure 10, Supplementary Tables 19, 20).

### Central aortic reflection magnitude (CARM)

**Figure 11:**
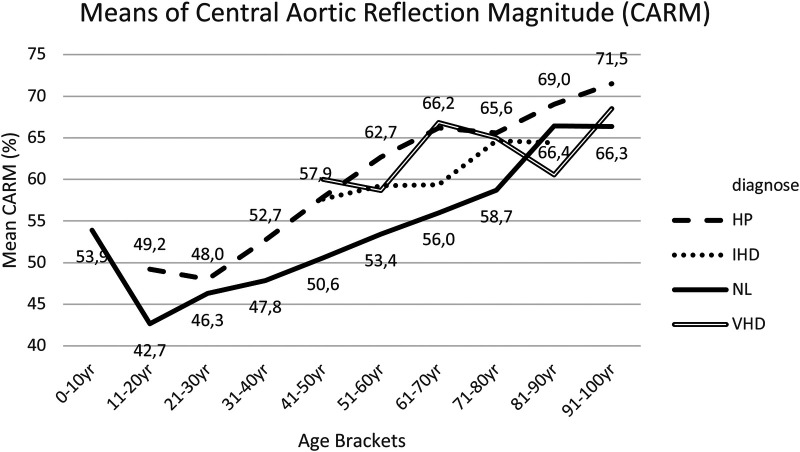
Mean CARM for each age bracket within NL and HP groups. NL, normal healthy subjects, HP, hypertensive patients; IHD, ischemic heart disease, VHD, valvular heart disease; CARM, central aortic reflection magnitude.

The results for *post hoc* comparisons indicated that the CARM mean score was significantly lower in group I (NL) than in group II (HP) in the following age brackets: 41–50 years, 51–60 years, 61–70 years and 71–80 years (Figure 11, Supplementary Table 21).

In group III IHD patients, compared with group I, CARM appeared to be higher within age brackets 41–50 years, 51–60 years, 61–70 years and 71–80 years, though not always having statistically significant variations (Figure 11, Supplementary Table 22).

In group IV VHD patients, CARM measurements were approximately equivalent to the measurements in group II (HP) and higher compared to group I (NL) with statistical significance within age brackets 41–50 years, 51–60 years, 61–70 years and 71–80 years (Figure 11, Supplementary Table 23).

**Figure 12:**
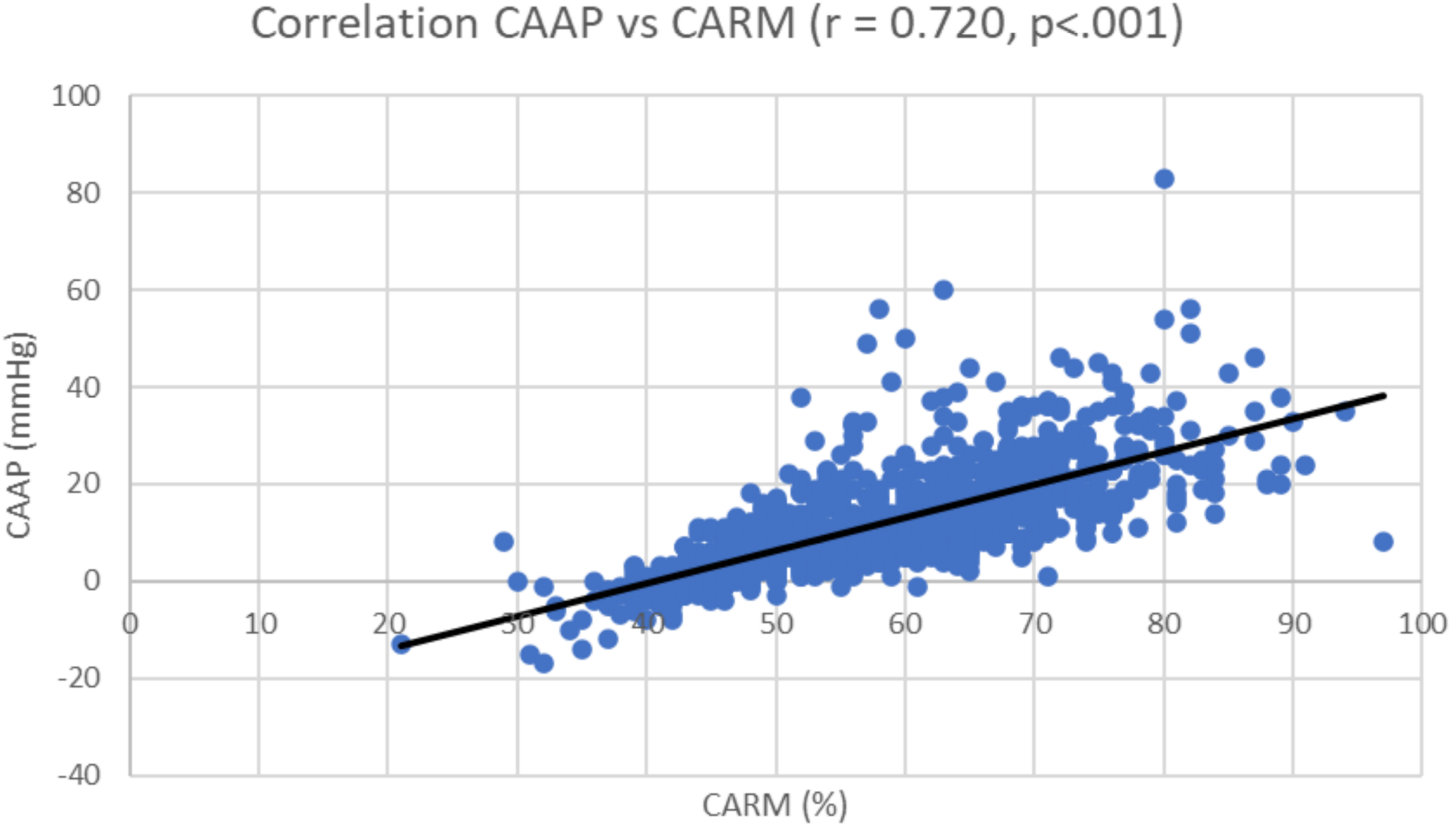
Correlation coefficient; central aortic augmented pressure (CAAP) vs. central aortic reflection magnitude (CARM).

Within this study there was a strong positive correlation between CAAP and CARM (Figure 12).

## Discussion

1. Overall, elevated blood pressure increases pulsatile aortic wall stress, consequently accelerating elastic degradation. Hypertension is viewed as an accelerated form of vascular aging that leads to aortic stiffening. Stiff arteries result in reduced diastolic pressure due to less elastic recoil to support diastolic pressure. This study consequently remained focused upon CABPS.

2. Aortic PWV is a non-invasive measure of aortic stiffness and arterial aging. Increase in PWV results in reflected pressure waves that return to the heart earlier in the cardiac cycle, which can augment the net central systolic pressure and concomitantly attenuate net central diastolic pressure, thereby increasing cardiac workload and decreasing coronary perfusion.

Within all four groups, this study noted a progressive increase in aortic PWV with age. Following the age of 50 years, the increase in PWV appeared more pronounced, especially in the hypertensive group (HP), resulting in distinct statistically significant variations between groups I (NL) and II (HP). These findings suggest—within elder age brackets—a less-compliant aorta with increased rapidity and earlier return of the reflected wave (13). Such data indicated that aortic PWV could be a more sensitive measurement of arterial aging in patients >50 years of age (14). Based on findings in the ischemic heart disease group (IHD), possibly, PWV is an independent predictor of coronary multi-vessel disease, although increased blood pressure, compared to the NL group, could also be a causative factor (15).

3. Concerning cases of decreased arterial elasticity and increased arterial stiffness, the reflected pressure wave in the arterial system becomes more important and increases central aortic systolic blood pressure. Conversely, within age brackets, this study observed a decrease in central aortic diastolic blood pressure, resulting in an increased CAPP reading. Increased CAPP is accompanied by increase in peripheral artery stiffness, which can lead to microvascular endothelial dysfunction, including coronary angiopathy and structural brain diseases (18). Elevated CAPP increases systolic pressure and contributes heavily to the development of predominant or isolated systolic hypertension. Furthermore, aortic stiffness, as indicated by elevated CAPP, might be viewed as an inevitable accompaniment of isolated systolic hypertension (19). Data suggested that even in very old, frail individuals, measurement of central aortic pulse pressure and arterial stiffness remain determinants of cognitive decline, morbidity and mortality.

Central aortic pulse pressure essentially appears to have considerable clinical value as an independent predictor of cardiovascular disease and mortality (30).

4. CAAP represents the effect of wave reflection upon the aortic pressure wave-form, being the difference between the two pressure peaks during ventricular systole. Superposition of forward and reflected pressure waves results in specific amounts of augmented pressure, with a characteristic inflection point located to the left of pressure peak in elderly individuals. Within younger individuals, the inflection point is typically found to the right-hand-side of pressure peaks, which results in negative values of augmented pressure (20).

The linear increase of CAAP with age suggests an increased wave refection, and—more specifically—an increase in the magnitude of wave refection.

Concerning the results of the 14 ADHD-patients, emotional factors undoubtedly play a role, though even following repeated reassuring measurements, similar results were always noted. Consequently, this study postulated whether hormonal factors or neurotransmitters (such as dopamine or noradrenaline) could influence CAAP pulse wave analysis measurements.

This study also probed if issues of “early vascular aging” (outlier +12 mmHg) and “super-normal vascular aging” (outlier −19 mmHg) could be taken into account (21).

5. Age, central aortic pulse pressure and isolated systolic hypertension are powerful determinants of cardiovascular risk, morbidity and mortality, due to increased arterial stiffness. The importance of increased arterial stiffness was further demonstrated by the observations that PWV and CAAIx are independent predictors of cardiovascular morbidity and mortality. CAAIx is defined as the reflected wave amplitude divided by pulse pressure, and expressed as a percentage. Large CAAIx readings indicate increased magnitude of wave reflection from the lower body or earlier return of the reflected wave towards the heart, as a result of increased pulse wave velocity and a less-compliant aorta (22).

Changes in CAAIx were more prominent in younger individuals (<50 years), where shifts in aortic PWV were more marked in older individuals (>50 years). Consequently, CAAIx might be a more sensitive marker of arterial aging and stiffness in younger patients, with aortic PWV being more sensitive within individuals over 50 years of age.

6. CARM is the ratio of Reflected Pulse Height to Forward Pulse Height, expressed as a percentage. The initial wave moves towards the periphery (Forward wave), while the reflected wave behave as a single wave moving towards the heart (Backward wave). The reflected pressure wave causes an aortic pressure augmentation and enhances coronary blood supply. Conversely, an excess of aortic pressure augmentation increases the risk of cardiovascular disease (24). Increased aortic wave reflection causes reduction on the contraction velocity of the left ventricle and is a predictor of cardiovascular events, being also hypothesized to be a cofactor in heart failure pathophysiology (25).

Issues such as obesity and diabetes also affect CARM, and increase both forward and backward reflections, resulting in increased arterial stiffness (26).

Interestingly, within this study, there was a strong positive correlation between CAAP and CARM. A lower heart rate increases central aortic blood pressure through enhanced backward wave pressure. There was also an inverse relationship between heart rate and backward wave pressures, which could have been amplified by underlying central aortic stiffness (27). Therapy aimed at decreasing reflection magnitude would reduce arterial stiffness and be valuable in the prevention of cardiovascular events (28). Furthermore, within a multi-ethnic population, initially free of clinically evident cardiovascular disease, CARM was found to be independently associated with all-cause mortality (29).

## Study limitations

Despite intense researcher efforts, this study had some limitations. Patients had to meet strict criteria to be included in the study. Possible drug therapy was not a contra-indication for inclusion, although treatment with calcium antagonists, beta blockers, diuretics, ACE inhibitors, sartans and statins might influence pulse wave analysis and PWV measurements to some extent.

Although the middle age groups exhibit a substantial and homogeneous population, the first three and the final age group are comparatively less in size. Within these age groups, the numbers of VHD and IHD are borderline normal. Within these groups we are careful in drawing definite conclusions. In groups aged 50 and over, these diagnoses are more common, and the numbers are therefore higher, which support more valid statistical analysis.

We could also mention the rather limited number of examined subjects in the age group 0–10 years, notwithstanding, on the other hand, the clear conclusive findings.

## Conclusions

- Research probing arterial vascular wall stiffness through pulse wave analysis, PWV and CABPS reading appear to have clinical value. In the risk stratification of arterial stiffness, hypertension and especially central aortic blood pressure are primordial and determining factors.
- Pulse wave analysis and PWV measurements do not yield definitive and fixed results, though do concern variable values, depending upon lifestyles, hormonal changes, drug treatments and other therapeutic interventions. Pulse wave analysis and PWV are important parameters in the evaluation, monitoring and treatment of arterial hypertension.
- Cause for concern were the elevated values for CAAP, CAAIx and CARM in the youngest age bracket 0–10 years. Furthermore, clear differences were established between non-ADHD and ADHD patients, with even a suspicion of variations between ADHD patients, depending on whether it mainly concerned an issue of attention deficit or hyperactivity disorder. Central aortic augmented pressure could be an important parameter in the diagnosis of ADHD. In this context, the results and findings within ADHD patients warrant confirmation and further investigation.
- The measured parameters for arterial stiffness can be affected by stress/anxiety and possibly also by neuro-hormonal regulation, neurotransmitters and a disturbed balance between the ortho-sympathetic and parasympathetic nervous system.
- The “Early Vascular Aging” syndrome and “supernormal vascular aging” values at young age deserves further attention.
- In patients with ischemic heart disease and valvular heart disease, the divergent PWV and pulse wave analysis measurements, compared to NH group, could be mainly attributed to the simultaneously slightly increased blood pressure values, unrelated to underlying heart disease.

## Perspectives

Pulse wave analysis and pulse wave velocity measurements are of great value, not only in the diagnosis of arterial stiffness, though also in the evaluation and follow-up of drug treatments or other therapeutic initiatives to reduce arterial stiffness.

Striking measurements with elevated values for CAAP, CAAIx and CARM within the youngest age bracket (0–10 years) indicated clear differences between non-ADHD and ADHD patients, with even a suspicion of variations between such ADHD patients, depending on whether this mainly concerned an attention deficit or hyperactivity disorder issue. Within this context, more extensive research is necessary, partly due to the scarce number of subjects examined in this study.

## Data Availability

The raw data supporting the conclusions of this article will be made available by the authors, without undue reservation.

## References

[1] Kim HL Kim SH. Pulse wave velocity in ahterosclerosis. Front Cardiovasc Med. (2019) 6:41. 10.3389/fcvm.2019.00041 31024934 PMC6465321

[2] Yasmin Brown MJ. Similarities and differences between augmentation index and pulse wave velocity in the assessment of arterial stiffness. QJM. (1999) 92(10):595–600. 10.1093/qjmed/92.10.595 10627881

[3] Nichols WW Petersen JW Denardo SJ Christou DD. Arterial stiffness, wave reflection amplitude and left ventricular afterload are increased in overweight individuals. Artery Res. (2013) 7(3–4):222–9. 10.1016/j.artres.2013.08.001

[4] Kanaki AI Sarafidis PA Georgianos PI Kanavos K Tziolas IM Zebekakis PE Effects of low-dose atorvastatin on arterial stiffness and central aortic pressure augmentation in patients with hypertension and hypercholesterolemia. Am J Hypertens. (2013) 26(5):608–16. 10.1093/ajh/hps098 23449607

[5] Thorin-Trescases N de Montgolfier O Pinçon A Raignault A Caland L Labbé P Impact of pulse pressure on cerebrovascular events leading to age-related cognitive decline. Am J Physiol Heart Circ Physiol. (2018) 314(6):H1214–24. 10.1152/ajpheart.00637.2017 29451817 PMC6032083

[6] Lo Gullo A Giuffrida C Morace C Squadrito G Magnano San Lio P Ricciardi L Arterial stiffness and adult onset vasculitis: a systematic review. Front Med (Lausanne). (2022) 9:824630. 10.3389/fmed.2022.824630 35646970 PMC9133451

[7] Mădălina Zota I Stătescu C Andy Sascău R Roca M Anghel L Maștaleru A Acute and long-term consequences of COVID-19 on arterial stiffness-A narrative review. Life (Basel). (2022) 12(6):781. 10.3390/life12060781 35743812 PMC9224691

[8] Roman MJ Devereux RB Kizer JR Okin PM Lee ET Wang W High central pulse pressure is independently associated with adverse cardiovascular outcome the strong heart study. J Am Coll Cardiol. (2009) 54(18):1730–4. 10.1016/j.jacc.2009.05.070 19850215 PMC3164777

[9] Chirinos JA Zambrano JP Chakko S Veerani A Schob A Willens HJ Aortic pressure augmentation predicts adverse cardiovascular events in patients with established coronary artery disease. Hypertension. (2005) 45(5):980–5. 10.1161/01.hyp.0000165025.16381.44 15837821

[10] Sugawara J Hayashi K Tanaka H. Arterial path length for arterial stiffness: methodological consideration. Am J Hypertens. (2016) 29(11):1237–44. 10.1093/ajh/hpw075 27496168

[11] Reusz GS Bárczi A Dégi A Cseprekál O Kis É Szabó Á Distance measurement for pulse wave velocity estimation in pediatric age: comparison with intra-arterial path length. Atherosclerosis. (2020) 303:15–20. 10.1016/j.atherosclerosis.2020.04.026 32464365

[12] Laurent S Boutouyrie P. Arterial stiffness and hypertension in the elderly. Front Cardiovasc Med. (2020) 7:544302. 10.3389/fcvm.2020.544302 33330638 PMC7673379

[13] Díaz A Galli C Tringler M Ramírez A Cabrera Fischer EI. Reference values of pulse wave velocity in healthy people from an urban and rural argentinean population. Int J Hypertens. (2014) 2014:653239. 10.1155/2014/653239 25215227 PMC4158305

[14] Valencia-Hernández CA Lindbohm JV Shipley MJ Wilkinson IB McEniery CM Ahmadi-Abhari S Aortic pulse wave velocity as adjunct risk marker for assessing cardiovascular disease risk: prospective study. Hypertension. (2022) 79(4):836–43. 10.1161/HYPERTENSIONAHA.121.17589 35139665 PMC9148390

[15] Ben Ahmed H Allouche E Chetoui A Beji M Boudiche F Ouechtati W Relationship between arterial stiffness and the severity of coronary artery disease in acute coronary syndrome. Ann Cardiol Angeiol (Paris). (2021) 70(1):33–40. 10.1016/j.ancard.2020.11.006 33256951

[16] Kalkan S Efe S Tasar O Karabay CY Kirma C. Evaluation of aortic distensibility in patients with mitral valve prolapse using echocardiography and applanation tonometry. Turk Kardiyol Dern Ars. (2020) 48(8):739–45. 10.5543/tkda.2020.52988 33257614

[17] Imbalzano E Vatrano M Ghiadoni L Mandraffino G Dalbeni A Khandheria BK Arterial stiffness and mitral regurgitation in arterial hypertension: an intriguing pathophysiological link. Vascul Pharmacol. (2018) 111:71–6. 10.1016/j.vph.2018.10.007 30359778

[18] de Montgolfier O Thorin-Trescases N Thorin E. Pathological Continuum from the rise in pulse pressure to impaired neurovascular coupling and cognitive decline. Am J Hypertens. (2020) 33(5):375–90. 10.1093/ajh/hpaa001 32202623 PMC7188799

[19] Mitchell GF. Arterial stiffness and hypertension: chicken or egg? Hypertension. (2014) 64(2):210–4. 10.1161/HYPERTENSIONAHA.114.03449 24799614 PMC4185002

[20] Poleszczuk J Debowska M Dabrowski W Wojcik-Zaluska A Zaluska W Waniewski J. Subject-specific pulse wave propagation modeling: towards enhancement of cardiovascular assessment methods. PLoS One. (2018) 13. 10.1371/journal.pone.0190972 PMC5764332 29324835

[21] Nilsson PM. Early vascular aging in hypertension. Front Cardiovasc Med. (2020) 7:6. 10.3389/fcvm.2020.00006 32118044 PMC7011189

[22] McEniery CM Yasmin Hall IR Qasem A Wilkinson IB Cockcroft JR Normal vascular aging: differential effects on wave reflection and aortic pulse wave velocity: the Anglo-Cardiff collaborative trial (ACCT). J Am Coll Cardiol. (2005) 46(9):1753–60. 10.1016/j.jacc.2005.07.037 16256881

[23] Lechner I Reindl M Tiller C Holzknecht M Niederreiter S Mayr A Determinants and prognostic relevance of aortic stiffness in patients with recent ST-elevation myocardial infarction. Int J Cardiovasc Imaging. (2022) 38:237–47. 10.1007/s10554-021-02383-0 34476665 PMC8818631

[24] Desbiens L Fortier C Nadeau-Fredette A Madore F Hametner B Wassertheurer S Prediction of cardiovascular events by pulse waveform parameters: analysis of CARTaGENE. J. Am. Heart Assoc. (2022) 11(17):e02660. 10.1161/JAHA.122.026603 PMC9496446 36056725

[25] Bouwmeester TA van de Velde L Galenkamp H Postema PG Westerhof BE van den Born B-JH Association between the reflection magnitude and blood pressure in a multiethnic cohort: the healthy life in an urban setting study. J Hypertens. (2022) 40(11):2263–70. 10.1097/HJH.0000000000003256 35950966 PMC9553245

[26] Tran AH Kimball TR Khoury PR Dolan LM Urbina EM. Obese and type 2 diabetic youth have increased forward and backward wave reflections. Arterioscler Thromb Vasc Biol. (2021) 41(2):944–50. 10.1161/ATVBAHA.120.315317 33297750 PMC8102303

[27] Mthembu N Peterson VR Norton GR Sadiq E Kolkenbeck-Ruh A Naran R Proximal aortic stiffness modifies the relationship between heart rate and backward wave and hence central arterial pulse pressure. Front Cardiovasc Med. (2022) 9:971141. 10.3389/fcvm.2022.971141 36337883 PMC9626514

[28] Park CM Hughes AD Henein MY Khir AW. Mechanisms of aortic flow deceleration and the effect of wave reflection on left ventricular function. Front Physiol. (2020) 11:578701. 10.3389/fphys.2020.578701 33250774 PMC7676911

[29] Zamani P Jacobs DR Jr Segers P Duprez DA Brumback L Kronmal RA Reflection magnitude as a predictor of mortality: the multi-ethnic study of atherosclerosis. Hypertension. (2014) 64(5):958–64. 10.1161/HYPERTENSIONAHA.114.03855 25259746 PMC4192021

[30] Said MA Eppinga RN Lipsic E Verweij N van der Harst P. Relationship of arterial stiffness Index and pulse pressure with cardiovascular disease and mortality. J Am Heart Assoc. (2018) 7(2):e007621. 10.1161/JAHA.117.007621 29358193 PMC5850166

