# Supplementary material for: Pulse wave analysis measurements: important, underestimated and undervalued parameters in cardiovascular health problems
Source: Front Cardiovasc Med. 2023 Nov 2;10:1266258. doi: 10.3389/fcvm.2023.1266258 (PMC10653328; doi:10.3389/fcvm.2023.1266258)
Supplement: Supplementary file 1 [file Datasheet1.docx]

Table 1. Pairwise Comparisons of the Means of Cuff Brachial Blood Pressure Systolic in NL and HP per stage of age.

| CBBPS : NL-HP | Mean  Difference | Std error | p |
| --- | --- | --- | --- |
| Age group 0-10 years | / | / | / |
| Age group 11-20 years | 22.843^*^ | 5.909 | <.001 |
| Age group 21-30 years | 31.122* | 7.496 | <.001 |
| Age group 31-40 years | 25.955* | 5.983 | <.001 |
| Age group 41-50 years | 24.638* | 3.718 | <.001 |
| Age group 51-60 years | 30.268* | 2.335 | <.001 |
| Age group 61-70 years | 26.569* | 2.109 | <.001 |
| Age group 71-80 years | 29.578* | 2.336 | <.001 |
| Age group 81-90 years | 34.335* | 3.844 | <.001 |
| Age group 91-100 years | 24.833 | 11.708 | .102 |
|  |  |  |  |

Table 2. Pairwise Comparisons of the Means of Cuff Brachial Blood Pressure Diastolic in NL and HP per stage of age.

| CBBPD : NL-HP | Mean difference | Std error | p |
| --- | --- | --- | --- |
| Age group 0-10 years | / | / | / |
| Age group 11-20 years | 15.340* | 4.938 | .002 |
| Age group 21-30 years | 21.081* | 5.504 | <.001 |
| Age group 31-40 years | 12.094* | 4.393 | .006 |
| Age group 41-50 years | 9.845* | 2.730 | .002 |
| Age group 51-60 years | 8.962* | 1.715 | <.001 |
| Age group 61-70 years | 4.066* | 1.548 | .049 |
| Age group 71-80 years | 4.676* | 1.715 | .039 |
| Age group 81-90 years | 8.439* | 2.822 | .017 |
| Age group 91-100 years | -1.333 | 8.597 | 1.000 |

Table 3. Pairwise Comparisons of the Means of *Central Aortic Blood Pressure systolic* in NL and HP per stage of age.

| CABPS : NL - HP | Mean difference | Std error | p |
| --- | --- | --- | --- |
| Age group 0-10 years | / | / | / |
| Age group 11-20 years | 23.200 | 18.604 | .213 |
| Age group 21-30 years | 29.541 | 20.733 | .155 |
| Age group 31-40 years | 24.817 | 16.550 | .134 |
| Age group 41-50 years | 23.022 | 10.285 | .152 |
| Age group 51-60 years | 18.191* | 6.459 | .030 |
| Age group 61-70 years | 25.478* | 5.833 | <.001 |
| Age group 71-80 years | 27.890* | 6.462 | <.001 |
| Age group 81-90 years | 31.565* | 10.633 | .018 |
| Age group 91-100 years | 23.667 | 32.385 | 1.000 |

Table 4. Pairwise Comparisons of the Means of *Central Aortic Blood Pressure diastolic* in NL and HP per stage of age.

| CABPD : NL - HP | Mean difference | Std error | p |
| --- | --- | --- | --- |
| Age group 0-10 years | / | / | / |
| Age group 11-20 years | 16.560* | 5.074 | .001 |
| Age group 21-30 years | 22.727* | 5.655 | <.001 |
| Age group 31-40 years | 13.969* | 4.514 | .002 |
| Age group 41-50 years | 10.510* | 2.805 | .001 |
| Age group 51-60 years | 8.940* | 1.762 | <.001 |
| Age group 61-70 years | 4.319* | 1.591 | .040 |
| Age group 71-80 years | 3.458 | 1.763 | .300 |
| Age group 81-90 years | 8.449* | 2.900 | .022 |
| Age group 91-100 years | -2.333 | 8.833 | 1.000 |

Table 5. Pairwise Comparisons of the Means of *Central Aortic Blood Pressure systolic* in NL and IHD per stage of age.

| CABPS : NL – IHD | Mean difference | Std error | p |
| --- | --- | --- | --- |
| Age group 0-10 years | / | / | / |
| Age group 11-20 years | / | / | / |
| Age group 21-30 years | / | / | / |
| Age group 31-40 years | / | / | / |
| Age group 41-50 years | 9.782 | 4.700 | .226 |
| Age group 51-60 years | 4.614 | 2.946 | 0.705 |
| Age group 61-70 years | 11.893 | 2.318 | <.001 |
| Age group 71-80 years | 11.526 | 2.382 | .<.001 |
| Age group 81-90 years | 14.158* | 4.048 | .003 |
| Age group 91-100 years | / | / | / |

Table 6. Pairwise Comparisons of the Means of *Central Aortic Blood Pressure diastolic* in NL and IHD per stage of age.

| CABPD : NL – IHD | Mean difference | Std error | p |
| --- | --- | --- | --- |
| Age group 0-10 years | / | / | / |
| Age group 11-20 years | / | / | / |
| Age group 21-30 years | / | / | / |
| Age group 31-40 years | / | / | / |
| Age group 41-50 years | 2.534 | 4.700 | 1.000 |
| Age group 51-60 years | .063 | 2.551 | 1.000 |
| Age group 61-70 years | -2.895 | 2.008 | .897 |
| Age group 71-80 years | 1.547 | 2.063 | 1.000 |
| Age group 81-90 years | 4.684 | 3.505 | 1.000 |
| Age group 91-100 years | / | / | / |

Table 7. Pairwise Comparisons of the Means of *Central Aortic Blood Pressure systolic* in NL and VHD per stage of age.

| CABPS : NL – VHD | Mean difference | Std error | p |
| --- | --- | --- | --- |
| Age group 0-10 years | / | / | / |
| Age group 11-20 years | / | / | / |
| Age group 21-30 years | / | / | / |
| Age group 31-40 years | / | / | / |
| Age group 41-50 years | 0.593 | 12.581 | 1.000 |
| Age group 51-60 years | 5.138 | 5.216 | 1.000 |
| Age group 61-70 years | 8.093* | 3.044 | .048 |
| Age group 71-80 years | 5.595 | 2.460 | .143 |
| Age group 81-90 years | 4.426 | 3.997 | .090 |
| Age group 91-100 years | 0.333 | 8.8053 | 1.000 |

Table 8. Pairwise Comparisons of the Means of *Central Aortic Blood Pressure diastolic* in NL and VHD per stage of age.

| CABPD : NL – VHD | Mean difference | Std error | p |
| --- | --- | --- | --- |
| Age group 0-10 years | / | / | / |
| Age group 11-20 years | / | / | / |
| Age group 21-30 years | / | / | / |
| Age group 31-40 years | / | / | / |
| Age group 41-50 years | .034 | 10.896 | 1.000 |
| Age group 51-60 years | 4.033 | 4.517 | 1.000 |
| Age group 61-70 years | 2.855 | 2.636 | 1.000 |
| Age group 71-80 years | 3.707 | 2.130 | .493 |
| Age group 81-90 years | 1.376 | 3.461 | 1.000 |
| Age group 91-100 years | 6.833 | 6.974 | .982 |

Table 9. Pairwise Comparisons of the Means of *Pulse Wave Velocity* in NL and HP per stage of age.

| PWV : NL-HP | Mean difference | Std error | p |
| --- | --- | --- | --- |
| Age group 0-10 years | / | / | / |
| Age group 11-20 years | 1.174 | 1.819 | .519 |
| Age group 21-30 years | 1.693 | 2.028 | .404 |
| Age group 31-40 years | 1.683 | 1.618 | .299 |
| Age group 41-50 years | 2.074 | 1.006 | .237 |
| Age group 51-60 years | 1.935* | .632 | .013 |
| Age group 61-70 years | 2.501* | .570 | <.001 |
| Age group 71-80 years | 2.972* | .632 | <.001 |
| Age group 81-90 years | 5.065* | 1.040 | <.001 |
| Age group 91-100 years | 4.250 | 3.167 | .540 |

Table 10. Pairwise Comparisons of the Means of *Pulse Wave Velocity* in NL and IHD per stage of age.

| PWV : NL-IHD | Mean difference | Std error | p |
| --- | --- | --- | --- |
| Age group 0-10 years | / | / | / |
| Age group 11-20 years | / | / | / |
| Age group 21-30 years | / | / | / |
| Age group 31-40 years | / | / | / |
| Age group 41-50 years | .815 | 1.461 | 1.000 |
| Age group 51-60 years | 1.168 | .935 | 1.000 |
| Age group 61-70 years | 1.660 | .721 | .129 |
| Age group 71-80 years | 1.749 | .741 | .110 |
| Age group 81-90 years | 2.232 | 1.258 | .459 |
| Age group 91-100 years | / | / | / |

Table 11. Pairwise Comparisons of the Means of *Pulse Wave Velocity* in NL and VHD per stage of age.

| PWV : NL-VHD | Mean difference | Std error | p |
| --- | --- | --- | --- |
| Age group 0-10 years | / | / | / |
| Age group 11-20 years | / | / | / |
| Age group 21-30 years | / | / | / |
| Age group 31-40 years | / | / | / |
| Age group 41-50 years | .377 | 2.789 | 1.000 |
| Age group 51-60 years | .288 | 1.622 | 1.000 |
| Age group 61-70 years | .333 | .946 | 1.000 |
| Age group 71-80 years | 1.214 | .765 | .677 |
| Age group 81-90 years | 2.249 | 1.243 | .423 |
| Age group 91-100 years | 1.150 | 2.504 | 1.000 |

Table 12. Pairwise Comparisons of the Means of *Central Aortic Pulse Pressure* in NL and HP per stage of age.

| CAPP : NL - HP | Mean difference | Std error | p |
| --- | --- | --- | --- |
| Age group 0-10 years | / | / | / |
| Age group 11-20 years | / | / | / |
| Age group 21-30 years | / | / | / |
| Age group 31-40 years | / | / | / |
| Age group 41-50 years | 12.512* | 2.873 | <.001 |
| Age group 51-60 years | 18.891* | 1.804 | <.001 |
| Age group 61-70 years | 21.224* | 1.629 | <.001 |
| Age group 71-80 years | 19.644* | 1.805 | <.001 |
| Age group 81-90 years | 23.250* | 2.970 | <.001 |
| Age group 91-100 years | 26.000* | 9.046 | .012 |

Table 13. Pairwise Comparisons of the Means of *Central Aortic Pulse Pressure* in NL and IHD per stage of age.

| CAPP : NL - IHD | Mean difference | Std error | p |
| --- | --- | --- | --- |
| Age group 0-10 years | / | / | / |
| Age group 11-20 years | / | / | / |
| Age group 21-30 years | / | / | / |
| Age group 31-40 years | / | / | / |
| Age group 41-50 years | 7.231 | 3.474 | .226 |
| Age group 51-60 years | 5.103 | 2.223 | .131 |
| Age group 61-70 years | 9.064* | 1.713 | <.001 |
| Age group 71-80 years | 13.151* | 1.761 | <.001 |
| Age group 81-90 years | 9.474* | 2.991 | .010 |
| Age group 91-100 years | 26.000* | 9.046 | .012 |

Table 14. Pairwise Comparisons of the Means of *Central Aortic Pulse Pressure* in NL and VHD per stage of age.

| CAPP : NL - VHD | Mean difference | Std error | p |
| --- | --- | --- | --- |
| Age group 0-10 years | / | / | / |
| Age group 11-20 years | / | / | / |
| Age group 21-30 years | / | / | / |
| Age group 31-40 years | / | / | / |
| Age group 41-50 years | 9.644 | 6.629 | 6.629 |
| Age group 51-60 years | 9.220 | 3.855 | .102 |
| Age group 61-70 years | 11.014* | 2.250 | <.001 |
| Age group 71-80 years | 9.380* | 1.818 | <.001 |
| Age group 81-90 years | 3.550 | 2.954 | 1.000 |
| Age group 91-100 years | 6.500 | 5.952 | .825 |

Table 15. Pairwise Comparisons of the Means of *Central Aortic Augmented Pressure* in NL and HP per stage of age.

| CAAP : NL- HP | Mean difference | Std error | p |
| --- | --- | --- | --- |
| Age group 0-10 years | / | / | / |
| Age group 11-20 years | 6.680 | 4.395 | .129 |
| Age group 21-30 years | 3.419 | 4.899 | .485 |
| Age group 31-40 years | 8.366* | 3.910 | .033 |
| Age group 41-50 years | 7.195* | 2.430 | .019 |
| Age group 51-60 years | 10.681* | 1.526 | <.001 |
| Age group 61-70 years | 15.699* | 1.378 | <.001 |
| Age group 71-80 years | 13.835* | 1.527 | <.001 |
| Age group 81-90 years | 12.930* | 2.512 | <.001 |
| Age group 91-100 years | 16.333 | 7.652 | .099 |

Table 16. Pairwise Comparisons of the Means of Central Aortic Augmented Pressure in NL and IHD per stage of age.

| CAAP : NL- IHD | Mean difference | Std error | p |
| --- | --- | --- | --- |
| Age group 0-10 years | / | / | / |
| Age group 11-20 years | / | / | / |
| Age group 21-30 years | / | / | / |
| Age group 31-40 years | / | / | / |
| Age group 41-50 years | 5.898 | 3.528 | .569 |
| Age group 51-60 years | 3.391 | 2.211 | 752 |
| Age group 61-70 years | 4.552 | 1.740 | .054 |
| Age group 71-80 years | 6.224* | 1.788 | .003 |
| Age group 81-90 years | 3.263 | 3.038 | 1.000 |
| Age group 91-100 years | / | / | / |

Table 17. Pairwise Comparisons of the Means of *Central Aortic Augmented Pressure* in NL and VHD per stage of age.

| CAAP : NL- VHD | Mean difference | Std error | p |
| --- | --- | --- | --- |
| Age group 0-10 years | / | / | / |
| Age group 11-20 years | / | / | / |
| Age group 21-30 years | / | / | / |
| Age group 31-40 years | / | / | / |
| Age group 41-50 years | .898 | 9.443 | 1.000 |
| Age group 51-60 years | 6.606 | 3.915 | .551 |
| Age group 61-70 years | 6.802* | 2.285 | .018 |
| Age group 71-80 years | 3.681 | 1.846 | .279 |
| Age group 81-90 years | 2.674 | 3.000 | 1.000 |
| Age group 91-100 years | 2.083 | -2.083 | 1.000 |

Table 18. Pairwise Comparisons of the Means of *Central Aortic Augmentation Index* in NL and HP per stage of age.

| CAAIx : NL- HP | Mean difference | Std error | p |
| --- | --- | --- | --- |
| Age group 0-10 years | / | / | / |
| Age group 11-20 years | 22.500* | 5.262 | <.001 |
| Age group 21-30 years | 11.436* | 5.864 | .050 |
| Age group 31-40 years | 17.661* | 4.681 | <.001 |
| Age group 41-50 years | 11.978* | 2.909 | <.001 |
| Age group 51-60 years | 12.803* | 1.827 | <.001 |
| Age group 61-70 years | 13.833* | 1.650 | <.001 |
| Age group 71-80 years | 11.746* | 1.828 | <.001 |
| Age group 81-90 years | 6.818 | 3.007 | .142 |
| Age group 91-100 years | 10.167 | 9.160 | .802 |

Table 19. Pairwise Comparisons of the Means of *Central Aortic Augmentation Index* in NL and IHD per stage of age.

| CAAIx : NL- IHD | Mean difference | Std error | p |
| --- | --- | --- | --- |
| Age group 0-10 years | / | / | / |
| Age group 11-20 years | / | / | / |
| Age group 21-30 years | / | / | / |
| Age group 31-40 years | / | / | / |
| Age group 41-50 years | 13.049* | 4.206 | .012 |
| Age group 51-60 years | 5.858 | 2.636 | .159 |
| Age group 61-70 years | 4.998 | 2.074 | .097 |
| Age group 71-80 years | 3.771 | 2.132 | .463 |
| Age group 81-90 years | .105 | 3.622 | 1.000 |
| Age group 91-100 years | / | / | / |

Table 20. Pairwise Comparisons of the Means of *Central Aortic Augmentation Index* in NL and VHD per stage of age.

| CAAIx : NL- VHD | Mean difference | Std error | p |
| --- | --- | --- | --- |
| Age group 0-10 years | / | / | / |
| Age group 11-20 years | / | / | / |
| Age group 21-30 years | / | / | / |
| Age group 31-40 years | / | / | / |
| Age group 41-50 years | 5.424 | 11.258 | 1.000 |
| Age group 51-60 years | 10.049 | 4.667 | .189 |
| Age group 61-70 years | 7.698* | 2.724 | .029 |
| Age group 71-80 years | 2.849 | 2.201 | 1.000 |
| Age group 81-90 years | 7.355 | 3.576 | .240 |
| Age group 91-100 years | .083 | 7.206 | . 1.000 |

Table 21. Pairwise Comparisons of the Means of C*entral Aortic Reflection Magnitude* in NL and HP per stage of age.

| CARM : NL - HP | Mean difference | Std error | p |
| --- | --- | --- | --- |
| Age group 0-10 years | / | / | / |
| Age group 11-20 years | 6.573 | 4.090 | 0.108 |
| Age group 21-30 years | 1.581 | 4.562 | .729 |
| Age group 31-40 years | 5.121 | 3.642 | 0.160 |
| Age group 41-50 years | 7.195* | 2.263 | 0.009 |
| Age group 51-60 years | 9.378* | 1.421 | <.001 |
| Age group 61-70 years | 10.144* | 1.284 | <.001 |
| Age group 71-80 years | 6.836* | 1.422 | <.001 |
| Age group 81-90 years | 2.617 | 2.340 | 1.000 |
| Age group 91-100 years | 5.167 | 7.127 | 1.000 |

Table 22. Pairwise Comparisons of the Means of C*entral Aortic Reflection Magnitude* in NL and IHD per stage of age.

| CARM : NL - IHD | Mean difference | Std error | p |
| --- | --- | --- | --- |
| Age group 0-10 years | / | / | / |
| Age group 11-20 years | / | / | / |
| Age group 21-30 years | / | / | / |
| Age group 31-40 years | / | / | / |
| Age group 41-50 years | 7.049 | 3.286 | .193 |
| Age group 51-60 years | 5.807* | 2.059 | .029 |
| Age group 61-70 years | 3.350 | 1.621 | .234 |
| Age group 71-80 years | 5.943* | 1.665 | .002 |
| Age group 81-90 years | 2.000 | 2.830 | 1.000 |
| Age group 91-100 years | 5.167 | 7.127 | 1.000 |

Table 23. Pairwise Comparisons of the Means of C*entral Aortic Reflection Magnitude* in NL and VHD per stage of age.

| CARM : NL - VHD | Mean difference | Std error | p |
| --- | --- | --- | --- |
| Age group 0-10 years | / | / | / |
| Age group 11-20 years | / | / | / |
| Age group 21-30 years | / | / | / |
| Age group 31-40 years | / | / | / |
| Age group 41-50 years | 9.424 | 6.271 | .799 |
| Age group 51-60 years | 5.236 | 3.647 | .908 |
| Age group 61-70 years | 10.850* | 2.128 | <.001 |
| Age group 71-80 years | 6.297* | 1.720 | .002 |
| Age group 81-90 years | 5.871 | 2.794 | .215 |
| Age group 91-100 years | 2.167 | 5.630 | 1.000 |

Table 24. Descriptive statistics per age bracket and diagnosis

| Age Bracket | Diagnosis | N | N Males | N Females | Mean BMI |
| --- | --- | --- | --- | --- | --- |
| 0-10yr | NL | 14 | 8 | 6 | 17.26 |
|  | HN | 0 | / | / | / |
|  | VHD | 0 | / | / | / |
|  | IHD | 0 | / | / | / |
| 11-20yr | NL | 51 | 33 | 18 | 19.90 |
|  | HN | 5 | 3 | 2 | 31.14 |
|  | VHD | 0 | / | / | / |
|  | IHD | 0 | / | / | / |
| 21-30yr | NL | 43 | 23 | 20 | 23.1 |
|  | HN | 4 | 2 | 2 | 26.5 |
|  | VHD | 0 | / | / | / |
|  | IHD | 0 | / | / | / |
| 31-40yr | NL | 32 | 16 | 16 | 26.2 |
|  | HN | 7 | 4 | 3 | 30.4 |
|  | VHD | 0 | / | / | / |
|  | IHD | 0 | / | / | / |
| 41-50yr | NL | 52 | 27 | 25 | 31,40 |
|  | HN | 19 | 11 | 8 | 31.95 |
|  | VHD | 2 | 1 | 1 | 32,20 |
|  | IHD | 6 | 3 | 3 | 26.63 |
| 51-60yr | NL | 112 | 60 | 52 | 26.04 |
|  | HN | 49 | 31 | 18 | 29.46 |
|  | VHD | 4 | 2 | 2 | 30.10 |
|  | IHD | 14 | 9 | 5 | 27.59 |
| 61-70yr | NL | 90 | 52 | 38 | 25.85 |
|  | HN | 76 | 45 | 31 | 28.94 |
|  | VHD | 17 | 11 | 6 | 26.80 |
|  | IHD | 30 | 16 | 14 | 27.22 |
| 71-80yr | NL | 60 | 34 | 26 | 25.48 |
|  | HN | 86 | 44 | 42 | 28.54 |
|  | VHD | 30 | 12 | 18 | 28.14 |
|  | IHD | 41 | 23 | 18 | 28.57 |
| 81-90yr | NL | 13 | 6 | 7 | 24.75 |
|  | HN | 39 | 17 | 22 | 27.19 |
|  | VHD | 13 | 7 | 6 | 26.21 |
|  | IHD | 16 | 12 | 4 | 28.8 |
| 91-100yr | NL | 4 | 1 | 3 | 24.53 |
|  | HN | 2 | 1 | 1 | 30.40 |
|  | VHD | 3 | 2 | 1 | 25.70 |
|  | IHD | 0 | / | / | / |
